# Targeting the IL-1β/IL-1Ra pathways for the aggregation of human islet amyloid polypeptide in an ex vivo organ culture system of the intervertebral disc

**DOI:** 10.1038/s12276-019-0310-7

**Published:** 2019-09-25

**Authors:** Xinghuo Wu, Zhiwei Liao, Kun Wang, Wenbin Hua, Xianzhe Liu, Yu Song, Yukun Zhang, Shuhua Yang, Cao Yang

**Affiliations:** Department of Orthopaedics, Union Hospital, Tongji Medical College, Huazhong University of Science and Technology, Wuhan, 430022 China

**Keywords:** Pathogenesis, Molecular biology

## Abstract

Intervertebral disc degeneration (IDD) is characterized by excessive apoptosis of nucleus pulposus (NP) cells and hyperactive extracellular matrix (ECM) catabolism. Our previous studies revealed the relationship between human islet amyloid polypeptide (hIAPP) and NP cell apoptosis. However, the role of hIAPP aggregates in IDD has not yet been investigated. This study aimed to determine whether the accumulation of hIAPP aggregates promotes IDD progression. The aggregation of hIAPP increased in human NP tissues during IDD. The deposition of hIAPP aggravated the compression-induced IDD that promoted NP cell apoptosis and ECM degradation via IL-1β/IL-1Ra signaling in an ex vivo rat disc model. Moreover, neutralizing IL-1β augmented the protective effects of hIAPP overexpression by decreasing hIAPP aggregation in human NP cells. These results suggest that the aggregation of hIAPP promotes NP cell apoptosis and ECM degradation ex vivo and in vitro by disrupting the balance of IL-1β/IL-1Ra signaling.

## Back pain: Proteins under pressure

Intervertebral disc degeneration (IDD), is caused in part by a protein, hIAPP, that is secreted by the cells that make insulin, a blood sugar regulator. In IDD, the nucleus pulposus in disc degenerate, causing low back pain. Recent research linked formation of hIAPP aggregates with death of intervertebral disc cells. Given inflammation’s known role in IDD, Cao Yang at the Huazhong University of Science and Technology, Wuhan, China, and colleagues investigated how hIAPP and inflammation interact in IDD. Using a method they developed to model IDD by applying compression to cultured rat intervertebral disc cells, they showed that accumulation of hIAPP aggregates triggered inflammation and accelerated cell death. Blocking the inflammation decreased hIAPP aggregation and slowed IDD progression. Methods to disrupt hIAPP aggregation hold promise for treatment of IDD.

## Introduction

Low back pain (LBP) associated with intervertebral disc degeneration (IDD) is a common cause of disability worldwide; ~80% of people will suffer from LBP at some point in their lives^1^. Many factors, such as obesity, smoking and diabetes mellitus (DM), are thought to be involved in IDD progression^2,3^. However, the etiology and pathogenesis of IDD remain unknown. The intervertebral disc (IVD) functions as a buffering and conjunction organ for bearing mechanical loads and maintaining spinal stability; it is composed of the inner nucleus pulposus (NP) and the outer annulus fibrous (AF)^4^. IDD is characterized by the depletion of resident cells and the degradation of the extracellular matrix (ECM)^5^. An increase in the apoptosis level of NP cells contributes to excessive cell death, which accelerates the progression of IDD. Recent studies have reported that the amelioration of NP cell apoptosis exerts a therapeutic effect on the progression of IDD^6–8^. However, further research on the prevention and reversal of IDD progression is necessary.

Human islet amyloid polypeptide (hIAPP), a 37–amino acid polypeptide, has been confirmed to damage cells through the formation of aggregates^9,10^. Recent studies have revealed that the aggregation of hIAPP contributes to cell death by inhibiting autophagy, inducing oxidative stress, and promoting inflammatory cytokine release and cell membrane disruption^11–15^. IAPP is a normally soluble protein that is released by cells and can form insoluble toxic aggregates^11^. Previously, we showed that hIAPP expression is related to the development of IDD and that IAPP may regulate ECM metabolism and control the crosstalk between apoptosis and autophagy in NP cells^16,17^. Many studies have reported that hIAPP aggregates are toxic and result in islet cell apoptosis in vitro and in vivo. The aggregation of hIAPP has been identified in the pancreas and other extra-pancreatic tissues^17,18^. Previous studies have revealed that extracellular components, such as glycosaminoglycan, can accelerate the deposition of hIAPP, especially in an acidic environment^19,20^. NP tissues are abundant in the ECM, and a relatively low pH has been confirmed in NP tissues during IDD^21^. This suggests that hIAPP aggregates may play an important role in IDD progression. However, the aggregation and deposition of hIAPP in NP tissues during IDD has not yet been investigated.

Accumulating evidence suggests that inflammation plays a critical role in the pathogenesis of IDD^22–24^. The proinflammatory cytokine interleukin-1β (IL-1β) is highly expressed in degenerative IVD tissues and has been proposed to be a major mediator of inflammation in the progression of IDD^25^. The stimulation of IL-1β leads to a significant increase in the apoptotic rate in NP cells and AF cells^26^. Moreover, as a proinflammatory mediator, IL-1β regulates the production of various cytokines, chemokines, and matrix metalloproteinases (MMPs), as well as a disintegrin and metalloproteinase with thrombospondin motifs (ADAMTSs), resulting in inflammation and ECM degradation during IDD^8,26^. In degenerative disc tissues, IL-1β increases rapidly, while IL-1 receptor antagonist (IL-1Ra) decreases consistently^27,28^. An imbalance between IL-1β and IL-1Ra accelerates the progression of IDD to some degree. A recent study showed that hIAPP aggregates induce FAS upregulation, caspase activation, and cell apoptosis by stimulating the expression of IL-1β and reducing the release of IL-1Ra^29^. Moreover, IL-1β impaired IAPP processing, thereby potentiating the formation of aggregates and facilitating the activation of cell death signaling^29^. We surmise that IL-1β/IL-1Ra signaling may be involved in IDD by interacting with the deposition and aggregation of hIAPP.

As of now, the role of the deleterious hIAPP aggregates in IDD progression has not yet been investigated. We hypothesized that hIAPP aggregates may accelerate NP cell apoptosis and ECM degradation and may also be related to IL-1β/IL-1Ra signaling. To test this hypothesis, we conducted our experiments using tissue samples and an ex vivo rat disc model (Fig. S1). The deposition of hIAPP aggregates was more evident in IDD tissues compared to normal disc tissues. An ex vivo IVD organ culture model was designed to investigate the role of hIAPP aggregates in NP cells during compression-induced IDD. The aggregation of hIAPP was verified to be toxic to NP cells by inducing excessive IL-1β expression and cell apoptosis. Neutralizing IL-1β attenuated the detrimental effects of hIAPP aggregates in the rat disc model, while IL-1Ra neutralization enhanced the toxicity of hIAPP aggregates. Furthermore, the effects of hIAPP aggregates in NP cells were verified in vitro. The blockade of IL-1β signaling reduced the formation of hIAPP aggregates, while the inhibition of IL-1Ra facilitated the deposition of hIAPP and promoted ECM degradation and cell apoptosis.

## Materials and methods

### Collection of human NP tissues

The experimental protocols were approved by the Ethics Committee of Tongji Medical College, Huazhong University of Science and Technology. Written informed consent was obtained from all participants involved in our study. Degenerative human NP tissues were collected from patients who underwent disc excision and spinal fusion surgery for IDD, while normal human NP tissues were obtained from patients who underwent deformity correction surgery for scoliosis. Detailed information for each patient is listed in Supplemental Table 1. Human NP tissues were collected and immediately sectioned for use in various experiments. One section from each sample was fixed in 4% buffered formaldehyde (pH 7.4) and used for histological analysis. A second section was immediately immersed in RNAlater (Thermo Fisher Scientific, Waltham, MA) and frozen in liquid nitrogen for protein and RNA analyses. A third section was placed in a sterile tube for cell isolation.

### Isolation and culture of NP cells

Isolated NP tissues were cut into pieces and enzymatically digested in 0.2% type II collagenase and 0.25% trypsin (Gibco Life Technologies, Paisley, UK) for 3 h. After being filtered and washed in PBS, the suspension was centrifuged, and the isolated cells were cultured in Dulbecco’s modified Eagle medium (DMEM) containing 15% fetal bovine serum (Gibco Life Technologies, Paisley, UK) and 1% penicillin/streptomycin (Thermo Fisher Scientific, Waltham, MA). The cells were incubated at 37 °C under 5% O_2_ to simulate the physiologically hypoxic disc environment. The culture medium was replaced twice each week, and the cells from the second or third passage were prepared for the following experiments.

### Ex vivo IVD organ culture model

IVDs were collected from Sprague-Dawley rats (400 g, 10 weeks old) with the ethical approval of the Animal Experimentation Committee of Huazhong University of Science and Technology. Caudal discs with complete endplates were isolated and cultured in DMEM containing 15% fetal bovine serum and 1% penicillin/streptomycin (Thermo Fisher Scientific, Waltham, MA). The osmolarity of the culture medium was adjusted to 400 mOsm, and physiological conditions were approximated by the addition of 1.5% of a 5 M NaCl and 0.4 M KCl solution. Samples were incubated under a hypoxic atmosphere (37 °C, 5% O_2_) with saturated humidity. The culture medium was replaced twice each week.

### Compression treatment

IVD organs or NP cells were cultured in a compression apparatus with a pressure of 1.0 MPa, as described previously^30,31^. The tissues or cells were placed in cell culture plates in a container (37° C) of double-distilled water to maintain a humidified atmosphere. After mixed compressed air was pumped into the compression apparatus, it was applied to deliver 1.0 MPa static compression for 2 weeks.

### Histological staining

The NP tissues or rat discs were washed in PBS, fixed with buffered formaldehyde (4%, pH 7.4) for 12 h, decalcified in a 10% formic acid solution, dehydrated by ethanol, and embedded in paraffin. The specimens were sectioned at 4 µm, and the sections were deparaffinized, rehydrated, and stained with hematoxylin and eosin (HE), safranin-O/fast green, Masson’s trichrome or alcian blue. The histological grades of the specimens were assessed according to standards established previously^32^.

### Immunofluorescence analysis

Immunofluorescence analysis was performed, as previously described^16,17^. Briefly, NP cells or tissues attached to slides were fixed with 4% paraformaldehyde and then permeabilized with 0.2% Triton X-100 (Beyotime Biotechnology, China) in PBS. The slides were washed in PBS, blocked with 2% bovine serum albumin in PBS for 2 h at 37 °C and then incubated with primary antibodies against caspase-3 (1:100; Cell Signaling Technology, Danvers, MA), caspase-9 (1:100; Cell Signaling Technology, Danvers, MA), IL-1β (1:100; Abcam, Cambridge, UK), IL-1Ra (1:100; Abcam, Cambridge, UK), COL2A1 (1:100; Abcam, Cambridge, UK), and FAS (1:150; Proteintech Group, China). After being washed twice, the slides were subsequently treated with a secondary goat anti-rabbit antibody (Boster Biological Technology, China) at 37 °C for 2 h. Nuclei were costained for 5 min with 0.1 g/mL 4-6-diamidino-2-phenylindole (Beyotime Biotechnology, China). Images were captured under a microscope (Olympus, BX53; Melville, NY, USA).

### TUNEL staining and thioflavin S staining

Terminal deoxynucleotidyl transferase dUTP nick end labeling (TUNEL) staining was used to assess cell apoptosis. Cells were fixed in 4% paraformaldehyde for 1 h and then treated with 0.5% Triton X-100 for 10 min. After being washed with PBS, the cells were incubated with reagents from a TUNEL reaction kit (Roche, Basel, Switzerland) according to the manufacturer’s instructions. Thioflavin S (Th-S) staining was used to evaluate the deposition of hIAPP aggregates. After being washed with PBS, the fixed cells were incubated with Th-S solution (Sigma-Aldrich, Germany). All of the images were captured under a fluorescence microscope (Olympus, BX53; Melville, NY, USA).

### Quantitative reverse transcription PCR

Total RNA was extracted from NP cells with Trizol reagent (Thermo Fisher Scientific, Waltham, MA) and then reverse-transcribed and amplified by RT-qPCR according to standard protocols. The sequences of the primers used for RT-qPCR are listed in Supplemental Table 2. Homo β-actin was used for normalization. All of the data were tested in triplicate.

### Western blot analysis

Proteins were isolated from cells or tissues using a Western cell lysis kit (Beyotime) according to the manufacturer’s instructions. The procedures and analysis were performed using standard protocols^16^. The following antibodies were used: hIAPP (1:800; Abcam, Cambridge, UK), FAS (1:2000; Proteintech Group, China), FASL (1:1000; OriGene Technologies GmbH), Bax (1:5000; Proteintech Group, China), Bcl-2 (1:2000; Proteintech Group, China), VDAC1 (1:2000; Abcam, Cambridge, UK), cyto-c (1:10,000; Abcam, Cambridge, UK), cleaved caspase-3 (1:1000; Cell Signaling Technology, Danvers, MA), cleaved caspase-9 (1:1000; Cell Signaling Technology, Danvers, MA), MMP3 (1:1000; Proteintech Group, China), MMP9 (1:800; Proteintech Group, China), MMP13 (1:800; Proteintech Group, China), ADAMTS5 (1:200; Proteintech Group, China), SOX9 (1:2000; Proteintech Group, China), aggrecan (1:800; Proteintech Group, China), COL2A1 (1:200; Abcam, Cambridge, UK) and GAPDH (1:1000; Proteintech Group, China). Horseradish peroxidase-conjugated secondary antibodies (Proteintech Group, China) were used, and the protein bands were visualized and detected using an enhanced chemiluminescence system. GAPDH was used as a loading control. The experiment was performed in triplicate.

### Enzyme-linked immunosorbent assay (ELISA)

The cell culture supernatant was collected and centrifuged at 1000 × *g* for 20 min to remove dead cells and debris. The IL-1β or IL-1Ra content in the supernatant was analyzed using an ELISA kit for IL-1β or IL-1Ra (Elabscience Biotechnology, Houston, TX, USA) according to the standard protocol. The experiment was performed in triplicate.

### Overexpression and knock-down experiments

NP cells were seeded and cultured in six-well plates and reached 70–80% confluence before transfection. For hIAPP overexpression, NP cells were infected with pLVX-mCMV-ZsGreen-PGK-Puro or pLVX-mCMV-ZsGreen-PGK-Puro-hIAPP according to the manufacturer’s instructions. For shRNA knockdown, three shRNA plasmids were designed, and their efficiencies were tested (Fig. S2). To exclude off-target effects, three shRNA plasmids were tested in the NP cell experiments^33^. The shRNA sequences are listed in Supplemental Table 3. Nontargeting shRNA was used as a negative control. Transgenic expression in NP cells was detected using RT-qPCR and Western blotting 48 h after transfection. The transfection efficiency was quantified by assessing ZsGreen-positive cells under a fluorescence microscope (Olympus, BX53; Melville, NY, USA).

### Statistical analysis

All experiments were performed independently at least in triplicate, and the data are presented as the mean ± standard deviation (SD). Student’s *t*-test and one-way or two-way analysis of variance (ANOVA) with Tukey’s *post hoc* test were used to assess the changes in the effects for each experimental group. Statistical significance was set to *P* < 0.05 and determined using GraphPad Prism 7 software (La Jolla, CA, USA).

## Results

### Human IAPP aggregates in degenerative intervertebral disc tissues

**Fig. 1:**
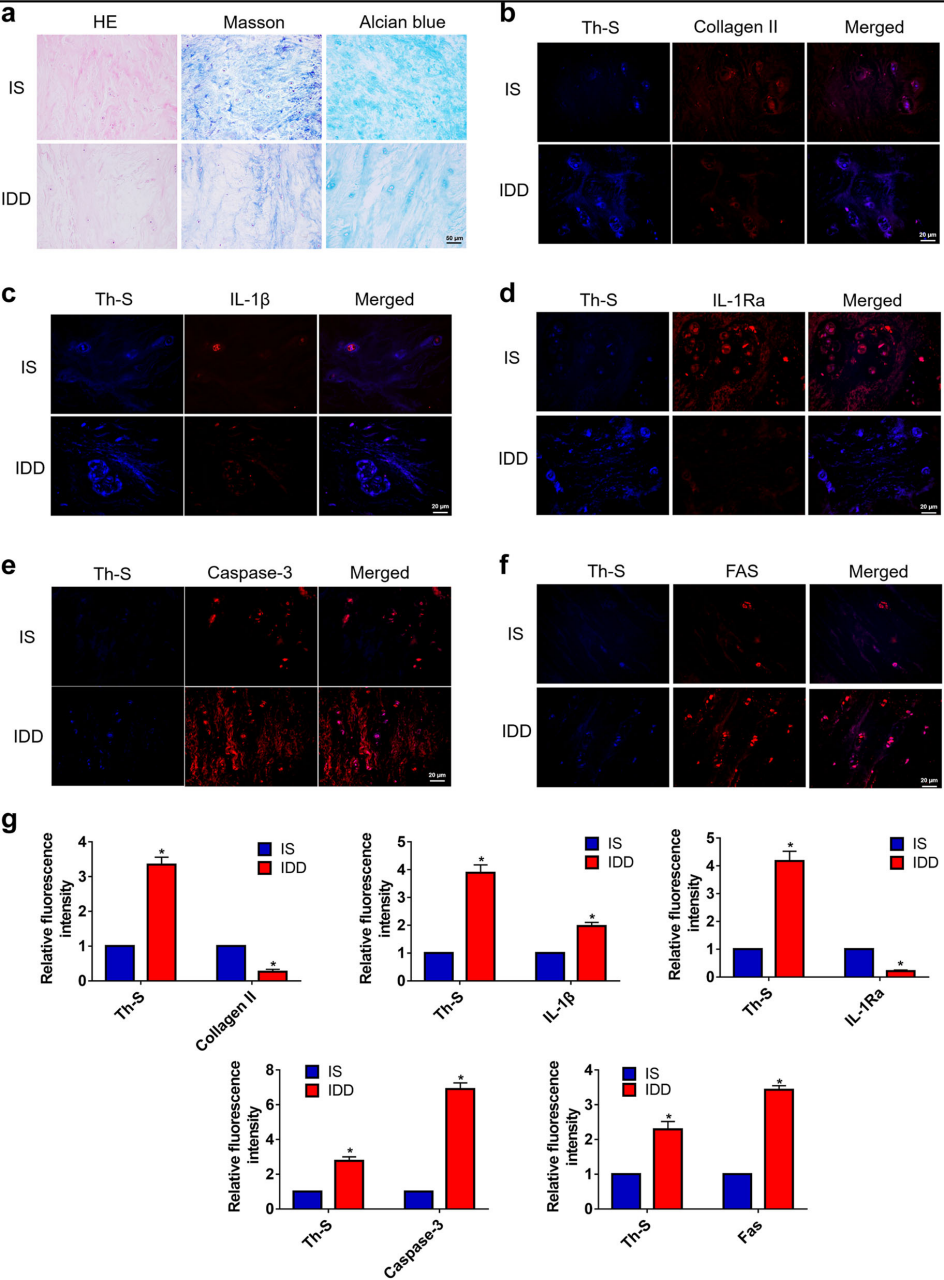
The aggregation of hIAPP in human NP tissues. **a** Representative images of histological staining of NP tissues from patients with idiopathic scoliosis (IS) or degenerative disc disease (IDD). (b-f) Immunofluorescence staining for collagen II **b**, IL-1β **c**, IL-1Ra **d** caspase-3 **e**, and FAS **f** and costaining with Th-S. Th-S staining showed the amyloidosis of hIAPP. **g** The quantitative analysis of the relative fluorescence intensity of Th-S staining and collagen II, IL-1β, IL-1Ra, caspase-3, and FAS immunofluorescence using Image-Pro Plus 6.0. The data are presented as the mean ± SD (*n* = 5). *P < 0.05 vs. the corresponding IS group

Histologic staining was used to evaluate the specimens from patients with scoliosis (IS) or IDD diseases. The results indicated that, compared to IS tissues, IDD tissues showed degenerative changes by HE, Masson’s, and alcian blue staining (Fig. 1a). To detect the aggregation of hIAPP, Th-S staining was used to stain hIAPP aggregates. Costaining with Th-S clearly showed that the expression of collagen II decreased as hIAPP aggregated in degenerative disc tissues (Fig. 1b). Meanwhile, the number of IL-1β-positive cells increased while the number of IL-1Ra-positive cells decreased in IDD (Fig. 1c, d). Given that IDD is characterized by the depletion of resident cells and an elevation in the apoptosis rate, the expression of caspase-3 and FAS were detected in IS and IDD tissues (Fig. 1e, f). The relative fluorescence intensity of Th-S, collagen II, IL-1β, IL-1Ra, caspase-3, and FAS were quantified accordingly (Fig. 1g). Taken together, these results indicated that hIAPP aggregated in degenerative intervertebral disc tissues and was associated with degenerative changes in NP tissues.

### Evaluation of hIAPP aggregation in an ex vivo model

**Fig. 2:**
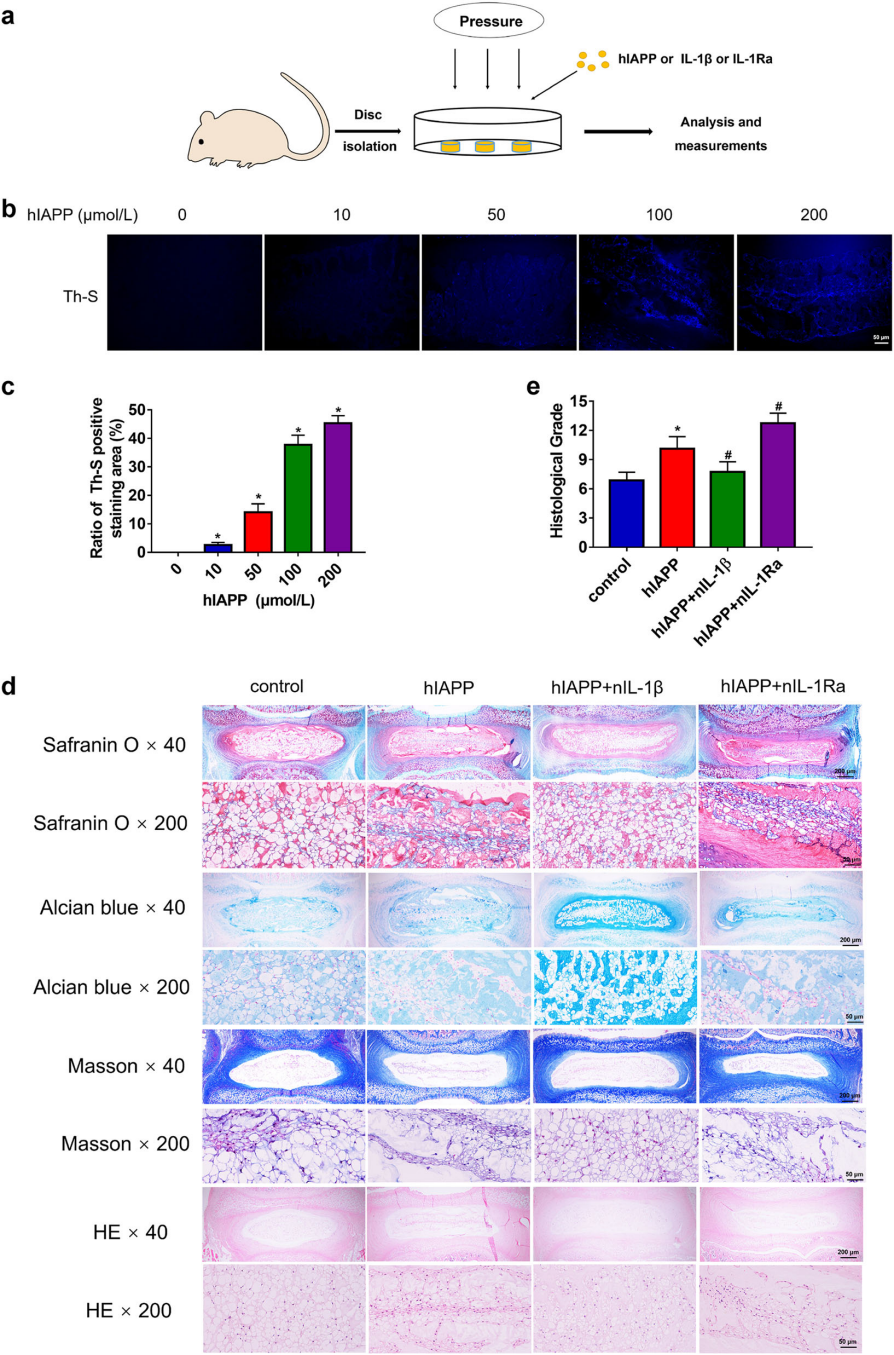
Histological staining of rat IVDs under compression and hIAPP treatment. **a** A schematic of the ex vivo culture and treatment. The rat IVDs were cultured ex vivo under compression treatment for two weeks. **b**, **c** Th-S staining of rat IVDs treated with 0, 10, 50, 100 or 200 μmol/L hIAPP **b** and the quantitative analysis of Th-S positive areas **c**. **d** Representative histological images of rat IVD tissues stained with safranin-O, alcian blue, Masson’s trichrome, and HE. The control group was treated with static compression for two weeks to induce IDD, and the other groups were treated with hIAPP (100 μmol/L), hIAPP and nIL-1β (1 μg/mL), or hIAPP and nIL-1Ra (1 μg/mL). **e** The histological grades of the rat IVD samples were evaluated. The grade of normal rat IVDs was 5. The data are presented as the mean ± SD (*n* = 8). *P < 0.05 vs. the control group. ^#^*P* < 0.05 vs^.^ the hIAPP group

**Fig. 3:**
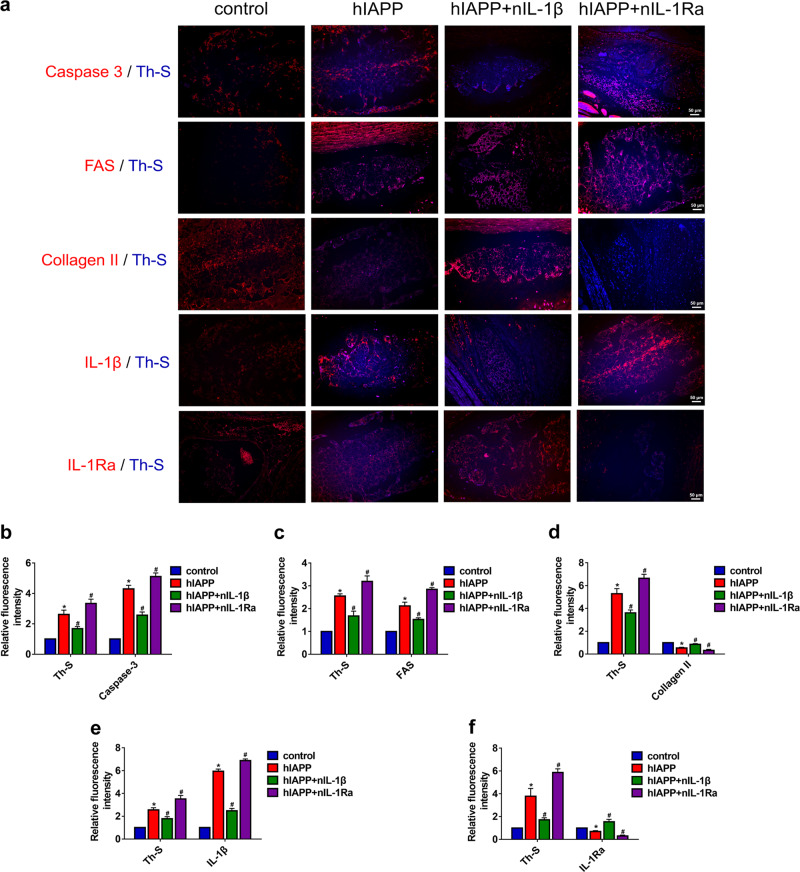
The aggregation of hIAPP in the rat ex vivo IDD disc model. The rat IVDs from all groups were cultured under compression treatment for two weeks. **a** Representative images of immunofluorescence for caspase-3, FAS, collagen II, IL-1β, and IL-1Ra (red) and costaining with Th-S staining (blue). **b–f** The quantitative analysis of the relative fluorescence intensity of Th-S staining and caspase-3 **b**, FAS **c**, collagen II **d**, IL-1β **e**, and IL-1Ra **f** immunofluorescence using Image-Pro Plus 6.0. The data are presented as the mean ± SD (*n* = 3). *P < 0.05 vs. the control group. ^#^*P* < 0.05 vs^.^ the hIAPP group

To evaluate the effects of hIAPP aggregates on IDD, we used an ex vivo model of IVD organ culture. rat discs were treated with exogenous hIAPP (0, 10, 50, 100, and 200 μmol/L) and cultured in a compression apparatus to induce IDD progression (Fig. 2a). Th-S staining of IVDs indicated that hIAPP aggregates were deposited in a concentration-dependent manner during compression-induced IDD (Fig. 2b, c). After two weeks of compression treatment, the discs were harvested and evaluated by histological staining (Fig. 2d, e). Th-S staining was performed, and the levels of caspase-3, FAS, collagen II, IL-1β, and IL-1Ra expression were measured by immunofluorescence staining (Fig. 3a). The blockade of IL-1β with nIL-1β reduced the formation of hIAPP aggregates and decreased caspase-3 and FAS expression (Fig. 3b, c). Moreover, the collagen II content increased in the hIAPP group cotreated with nIL-1β (Fig. 3d). The effects of nIL-1β and nIL-1Ra on the expression of IL-1β and IL-1Ra were both verified (Fig. 3e, f). These results demonstrated that the deposition of hIAPP aggravated IDD development, while cotreatment with nIL-1β ameliorated this effect.

### Effects of hIAPP aggregates on ECM remodeling and cell apoptosis in an ex vivo model

**Fig. 4:**
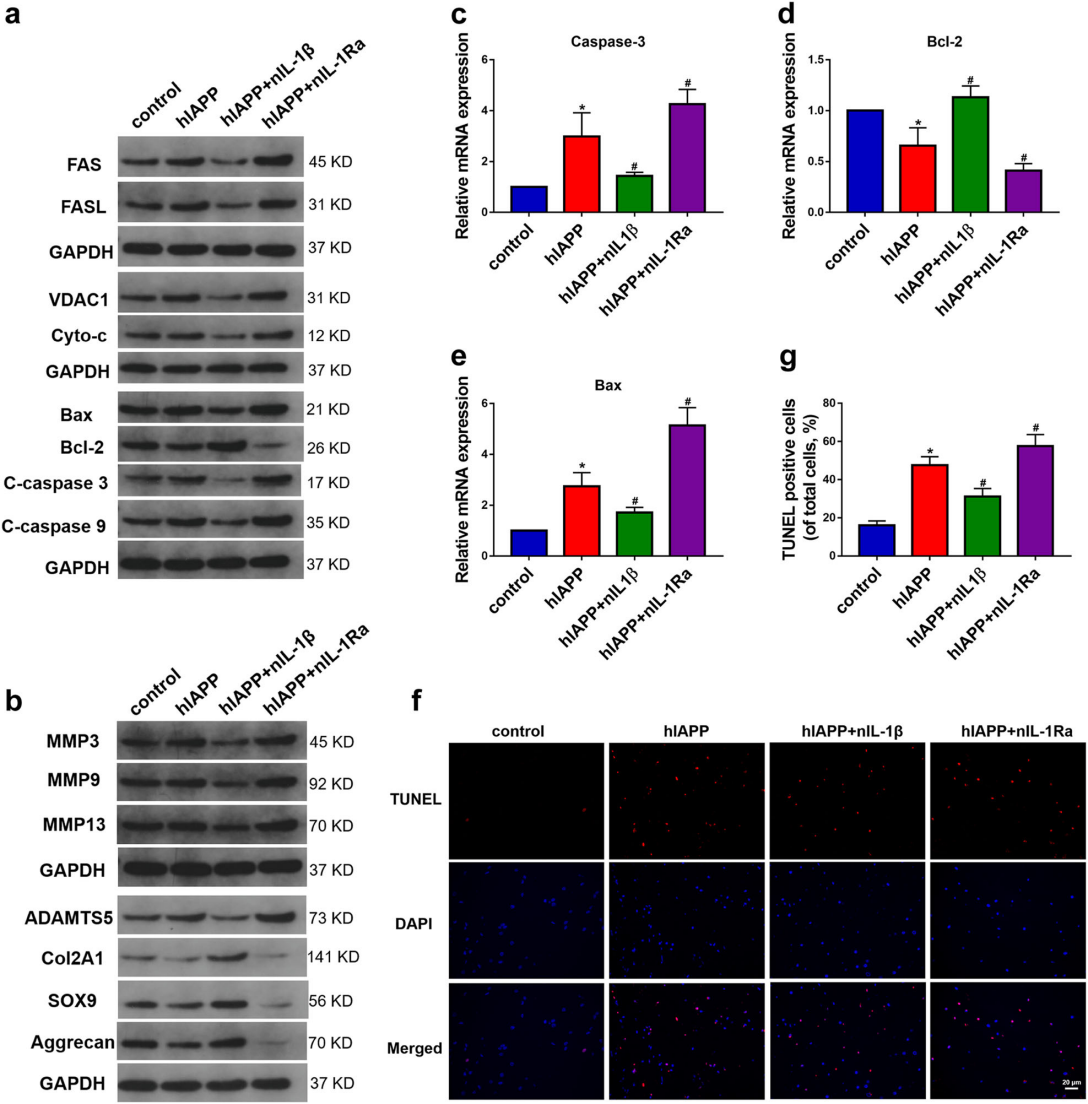
The effects of hIAPP aggregation on cell apoptosis and ECM metabolism ex vivo. **a** Western blot analysis was used to evaluate the protein expression of FAS, FASL, VDAC1, Cyto-c, Bax, Bcl-2, cleaved caspase-3, and caspase-9. GAPDH was used as an internal control. **b** Western blot analysis was used to evaluate the protein expression of MMP3, MMP9, MMP13, ADAMTS5, COL2A1, SOX9, and aggrecan. GAPDH was used as an internal control. **c–e** The mRNA expression levels of Bax **c**, Bcl-2 **d** and caspase-3 **e** were measured by RT-qPCR. β-actin was used as an internal control. **f–g** Representative images of TUNEL analysis **f** and the quantitative analysis of the TUNEL-positive cell rate **g**. The data are presented as the mean ± SD (*n* = 3). *P < 0.05 vs. the control group. ^#^*P* < 0.05 vs^.^ the hIAPP group

To further investigate the effects of hIAPP aggregates on IDD, the levels of proteins involved in ECM metabolism and cell apoptosis were measured by Western blot analysis (Fig. 4a, b). hIAPP deposition promoted the upregulation of FAS, the ligand of FAS (FASL), apoptosis-related proteins, voltage-dependent anion-selective channel 1 (VDAC-1), cytochrome C (cyto-C), and Bax (Fig. S3). The aggregation of hIAPP also decreased the level of the anti-apoptotic protein Bcl-2 and facilitated the cleavage of caspase-9 and caspase-3. The transcriptional levels of Bax, Bcl-2, and caspase-3 were evaluated by RT-qPCR analysis (Fig. 4c–e). Moreover, hIAPP aggregates promoted the expression of proteins involved in ECM catabolism and decreased the expression of proteins involved in ECM anabolism (Fig. S3). The TUNEL staining results also supported the assessment that hIAPP aggregates promoted cell apoptosis during the IDD process (Fig. 4f, g). These results revealed that hIAPP aggregates resulted in NP cell apoptosis and ECM catabolism and that these effects were ameliorated by treatment with nIL-1β and aggravated by nIL-1Ra.

### Effects of hIAPP overexpression upon cotreatment with IL-1β neutralizing antibodies on ECM remodeling and cell apoptosis in vitro

**Fig. 5:**
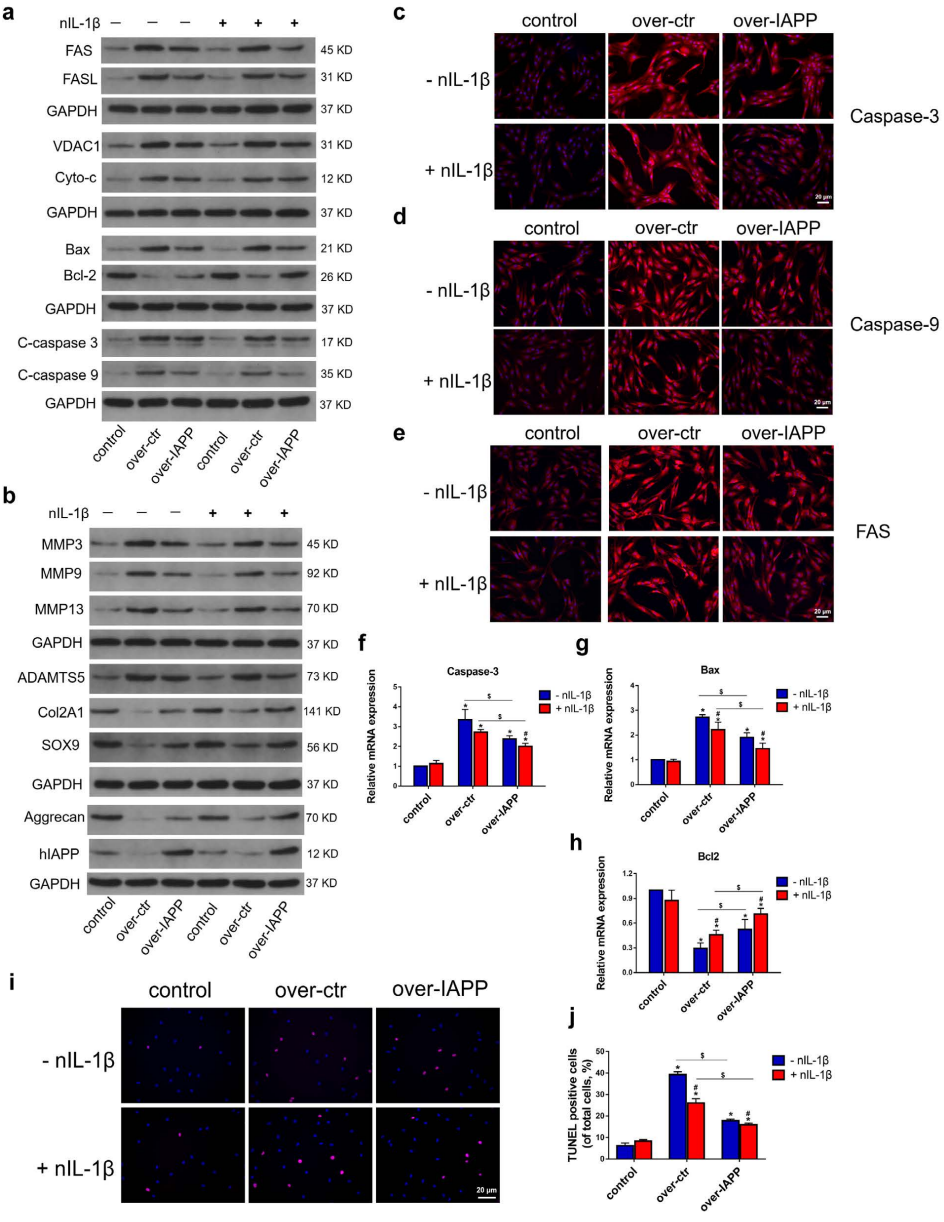
The effects of IL-1β neutralization on ECM remodeling and cell apoptosis in hIAPP-overexpressing NP cells. Human NP cells were transfected with pLVX-mCMV-Puro (over-ctr) and pLVX-mCMV-IAPP (over-IAPP) with or without nIL-1β (1 μg/mL) under static compression for one week. **a** Western blot analysis was used to evaluate the protein expression levels of FAS, FASL, VDAC1, Cyto-c, Bax, Bcl-2, cleaved caspase-3, and caspase-9. GAPDH was used as an internal control. **b** Western blot analysis was used to evaluate the protein expression levels of MMP3, MMP9, MMP13, ADAMTS5, COL2A1, SOX9, aggrecan, and hIAPP. **c–e** Representative images of immunofluorescence for caspase-3 **c**, caspase-9 **d**, and FAS **e**. **f–h** The mRNA expression levels of caspase-3 **f**, Bax **g**, and Bcl-2 **h** were measured by RT-qPCR. β-actin was used as an internal control. **i**–**j** Representative images of TUNEL analysis **i** and the quantitative analysis of the TUNEL-positive cell rate **j**. The data are presented as the mean ± SD (*n* = 3). *P < 0.05 vs. the control group. ^#^*P* < 0.05 vs^.^ the nIL-1β-untreated group. ^$^*P* < 0.05 vs. the corresponding group

A previous study demonstrated the beneficial effect of hIAPP overexpression in human AF cells^17^. In addition, the effect of neutralizing IL-1β on ECM remodeling and cell apoptosis was evaluated by Western blot analysis (Fig. 5a, b). Consistent with this previous study, the overexpression of hIAPP reduced the expression of apoptotic proteins and ECM degradation compared to those in the compression-treated control group. Interestingly, neutralizing IL-1β enhanced the beneficial effects of hIAPP overexpression by reducing the expression of pro-apoptotic proteins and proteins involved in ECM catabolism while preserving ECM components (Fig. S4). The expression of caspase-3, caspase-9 and FAS in the hIAPP overexpression group with or without nIL-1β treatment was evaluated by immunofluorescence analysis (Fig. 5c–e). RT-qPCR analysis also showed that cotreatment with nIL-1β reduced Bax and caspase-3 expression and promoted Bcl-2 expression (Fig. 5f–h). Moreover, the overexpression of hIAPP reduced the apoptosis rate according to the TUNEL results, and neutralizing IL-1β augmented these anti-apoptotic effects (Fig. 5i, j).

### Effects of hIAPP silencing upon cotreatment with IL-1Ra neutralizing antibodies on ECM remodeling and cell apoptosis in vitro

**Fig. 6:**
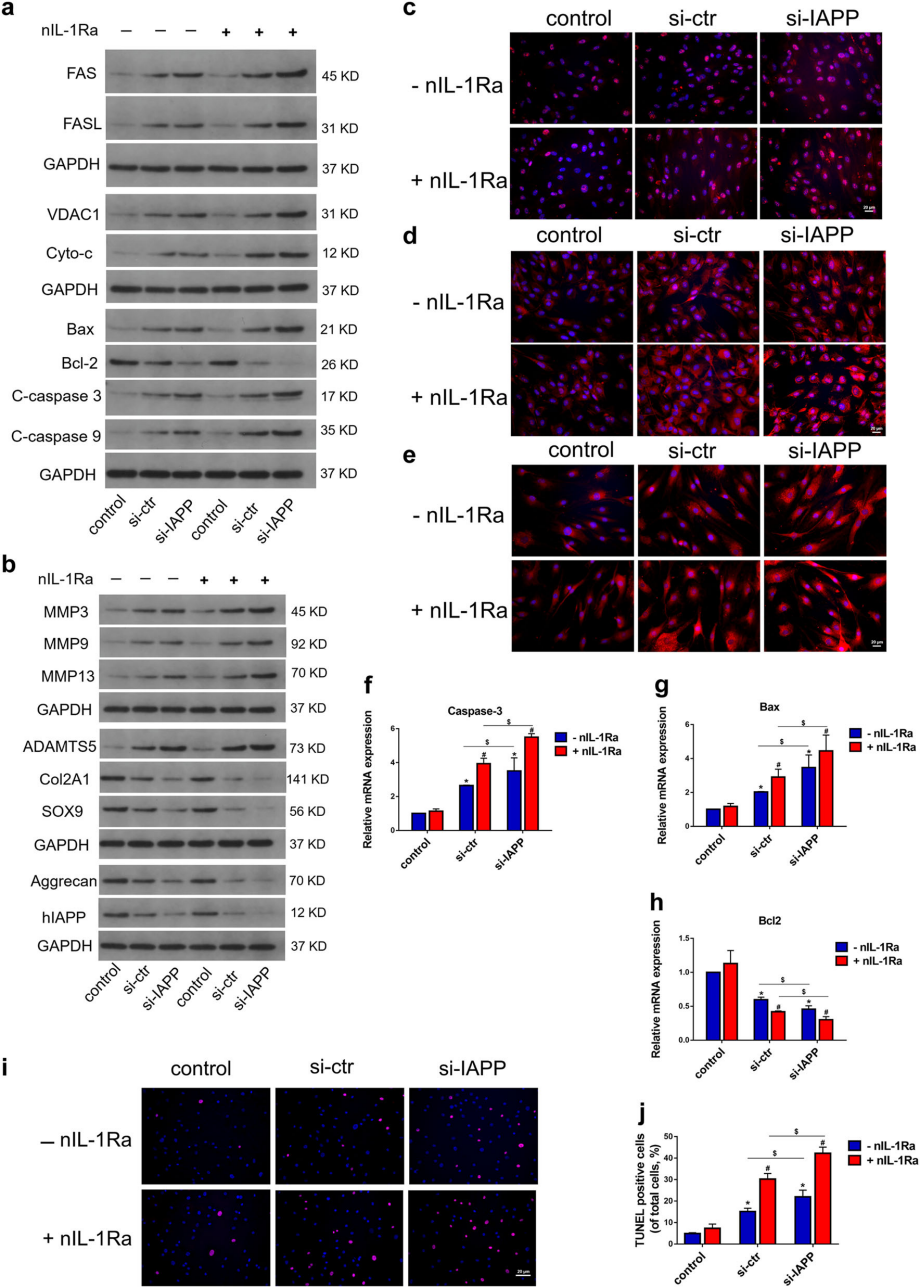
The effects of IL-1Ra neutralization on ECM remodeling and cell apoptosis in hIAPP-silenced NP cells. Human NP cells were transfected with PLVX-ShRNA2-Puro (si-ctr) and pLVX-ShRNA2-Puro-IAPP (si-IAPP) with or without nIL-1Ra (1 μg/mL) under static compression for one week. **a** Western blot analysis was used to evaluate the protein expression levels of FAS, FASL, VDAC1, Cyto-c, Bax, Bcl-2, cleaved caspase-3, and caspase-9. GAPDH was used as an internal control. **b** Western blot analysis was used to evaluate the protein expression levels of MMP3, MMP9, MMP13, ADAMTS5, COL2A1, SOX9, aggrecan, and hIAPP. **c–e** Representative images of immunofluorescence for caspase-3 **c**, caspase-9 **d**, and FAS **e**. **f–h** The mRNA expression levels of caspase-3 **f**, Bax **g**, and Bcl-2 **h** were measured by RT-qPCR. β-actin was used as an internal control. **i**, **j** Representative images of TUNEL analysis **i** and the quantitative analysis of the TUNEL-positive cell rate **j**. The data are presented as the mean ± SD (*n* = 3). *P < 0.05 vs. the control group. ^#^*P* < 0.05 vs^.^ the nIL-1Ra-untreated group. ^$^*P* < 0.05 vs. the corresponding group

The expression of proteins related to ECM remodeling and cell apoptosis in the hIAPP-silenced group cotreated with nIL-1Ra was measured by Western blot analysis (Fig. 6a, b). Neutralizing IL-1Ra promoted cell apoptosis via the upregulation of FAS, VDAC1, cyto-C, caspase-3, and Bax and facilitated the expression of MMPs and ADAMTSs (Fig. S5). The expression levels of caspase-3, caspase-9, and FAS were also evaluated by immunofluorescence analysis (Fig. 6c–e). The transcriptional levels of Bax and caspase-3 increased, and the expression of Bcl-2 decreased significantly in the nIL-1Ra-treated group (Fig. 6f–h). Compared to that in the hIAPP-silenced group, the ratio of TUNEL-positive cells increased significantly in the group cotreated with nIL-1Ra (Fig. 6i, j). These results showed that an imbalance in IL-1β/IL-1Ra signaling impacted ECM remodeling and cell apoptosis in hIAPP-overexpressing or hIAPP-knockdown human NP cells.

### An imbalance in IL-1β/IL-1Ra signaling promoted ECM degradation and cell apoptosis by regulating the deposition of hIAPP aggregates in human NP cells

**Fig. 7:**
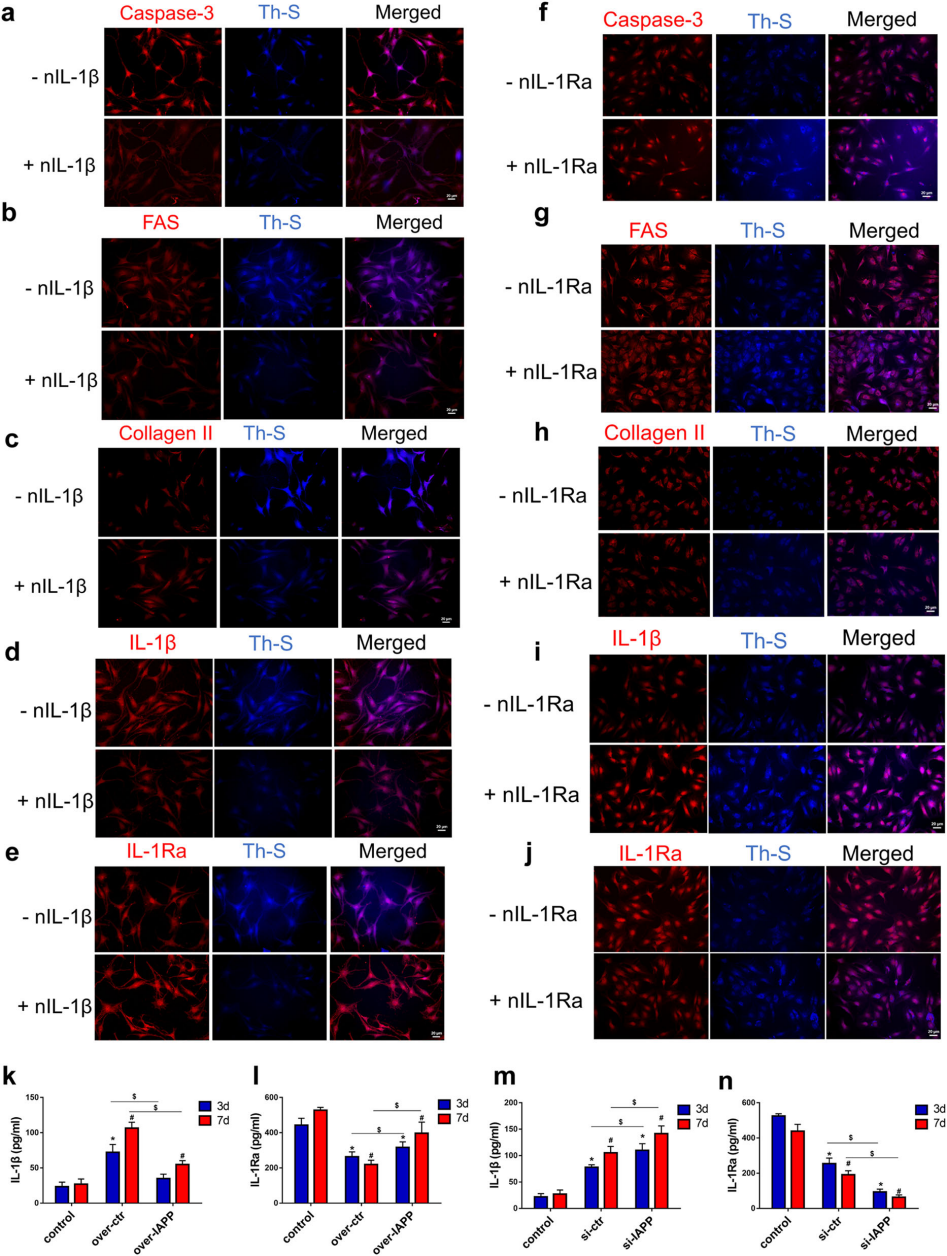
IL-1β/IL-1Ra signaling regulated ECM metabolism and cell apoptosis through the deposition of hIAPP aggregates in human NP cells. **a–e** Representative images of immunofluorescence for caspase-3 **a**, FAS **b**, collagen II **c**, IL-1β **d**, and IL-1Ra **e** and costaining with Th-S. hIAPP was overexpressed in human NP cells with or without nIL-1β (1 μg/mL) under static compression for one week. **f–j** Representative images of immunofluorescence for caspase-3 **f**, FAS **g**, collagen II **h**, IL-1β **i** and IL-1Ra **j** and costaining with Th-S. hIAPP was knocked down in human NP cells with or without nIL-1Ra (1 μg/mL) under static compression for one week. **k–n** The content of IL-1β and IL-1Ra in the culture supernatant was measured by ELISA kits in hIAPP-overexpressed **k**, **l** or hIAPP-silenced **m**, **n** NP cells under compression treatment for 3 d or 7 d. The data are presented as the mean ± SD (*n* = 3). *P < 0.05 vs. the 3-d control group. ^#^*P* < 0.05 vs^.^ the 7-d control group. ^$^*P* < 0.05 vs. the corresponding group

To determine whether an imbalance in IL-1β/IL-1Ra signaling influenced ECM metabolism and cell apoptosis via the aggregation of hIAPP, double immunofluorescence staining for hIAPP aggregates was used. Neutralizing IL-1β antibodies inhibited the immunoreactivity of IL-1β and promoted IL-1Ra expression. In hIAPP-overexpressing NP cells, neutralizing IL-1β reduced the expression of caspase-3 and FAS and decreased collagen II degradation (Fig. 7a–e). Interestingly, the number of hIAPP aggregates stained by Th-S decreased significantly when NP cells were cotreated with nIL-1β (Fig. S6). In contrast, neutralizing IL-1Ra aggravated the detrimental effects of hIAPP silencing on ECM remodeling and cell apoptosis as the number of hIAPP aggregates increased (Fig. 7f–j). The levels of IL-1β and IL-1Ra were measured by ELISA, and the IL-1β/IL-1Ra ratio was calculated accordingly (Fig. 7k–n). These results demonstrated that an imbalance in IL-1β/IL-1Ra altered the effects of hIAPP overexpression or silencing in human NP cells by regulating the formation of hIAPP aggregates.

## Discussion

IDD and related musculoskeletal disorders impose a great socioeconomic burden in countries throughout the world^34^. However, the pathogenic mechanisms of IDD remain unknown, and the search for effective treatments is ongoing. Our study demonstrated that toxic hIAPP was deposited and aggregated in degenerative IVD tissues and was closely related to the progression of IDD. hIAPP aggregates promoted NP cell apoptosis and regulated inflammation and ECM metabolism. The pro-inflammatory cytokine IL-1β promoted the aggregation and deposition of hIAPP. The blockade of IL-1β signaling reduced ECM degradation and cell apoptosis. Moreover, we designed a compression system to induce disc degeneration in an ex vivo culture model. The amyloidogenesis of hIAPP was enhanced during compression-induced NP cell degeneration. Neutralizing IL-1β facilitated the aggregation of hIAPP and augmented its detrimental effects. Enhancing IL-1β signaling by inhibiting IL-1Ra significantly promoted the formation of hIAPP aggregates (Fig. S7).

The ex vivo culture system in our study simulated the in vivo IVD environment. The IVD organ culture, including the intervertebral disc and adjacent endplates, allowed nutrients and outer substances to penetrate into the inner disc while leaving the IVD structure and native ECM intact^35–37^. Compared to in vitro studies, the ex vivo culture system, which utilized IVD explants, offers advantages for investigating the pathological mechanisms of IDD^38^. Mechanical stimulation was used to accelerate the progression of IDD, which was confirmed to be related to NP cell apoptosis and matrix degradation^39,40^. Previous studies have shown that a static or dynamic loading organ culture simulates cellular responses to mechanical loading of the IVD^38,41,42^. Consistent with Yurube’s study^41^, the upregulation of the expression of ECM catabolism enzymes (MMPs and ADAMTSs) and the downregulation of the expression of anabolism genes (ACAN, COL2A1, and SOX9) were observed in IDD in our compression-induced degeneration model. Meanwhile, pro-apoptotic proteins (FAS, Bax, VDAC1, cyto-C, and caspase-3) were increased and the anti-apoptotic protein Bcl-2 decreased in compression-induced degeneration. These results indicated that mechanical loading effectively induced IDD in this ex vivo model. Studies have shown that the structure of mouse IAPP is different from that of human IAPP and that mouse IAPP does not form toxic aggregates^43,44^. In an ex vivo rat model, exogenous hIAPP was applied to rat discs during compression-induced IDD. The disc samples from the exogenous hIAPP group showed a higher histological grade and obvious characteristics of degeneration.

Human IAPP is always deposited in matrix-abundant tissues and has been confirmed as the primary culprit in pancreatic cell loss^45^. Accumulating evidence has suggested that the oligomers rather than the monomers of hIAPP are responsible for cell apoptosis^11^. The mature monomer form of hIAPP has been found to be nontoxic to cells while prone to aggregation in T2D individuals; however, the underlying mechanism is currently unknown^11,46–48^. Our previous studies showed that the overexpression of hIAPP in human AF or NP cells inhibits apoptosis and that the downregulation of hIAPP expression induces the death of disc cells^16,17^. In the present study, the analysis of IVD tissue samples suggested that hIAPP deposited and aggregated in degenerative IVD tissues. In an ex vivo model, the catabolism- and apoptosis-related genes were upregulated, while ECM degradation and excessive NP cell apoptosis were observed to increase along with the number of hIAPP aggregates. These results demonstrated that the formation of toxic hIAPP aggregates might occur as a consequence or as a cause of IDD. Several studies have reported that hIAPP aggregates faster in acidic environments and maintains a soluble nontoxic form at a higher pH^20,44^. IDD has been confirmed to be related to an increase in lactic acid levels and the intradiscal pH^21,49^. This partly explains the significant deposition of hIAPP aggregates in IDD tissues. However, the underlying mechanisms of hIAPP aggregation in IVD tissues remain unclear and require further study.

The pathogenic mechanism of hIAPP aggregates has been confirmed to be closely related to the production of IL-1β^12^. A recent study showed that IL-1β plays a dual role in hIAPP-induced cell damage^29^. IL-1β impairs the processing of hIAPP, resulting in the aggregation of its immature form, which is easily deposited in human organs or tissues. Moreover, hIAPP aggregates also stimulate the expression of IL-1β, causing a vicious cycle. During the progression of IDD, the increased level of IL-1β expression has also been detected, and IL-1β has been confirmed as a critical mediator of IDD^50,51^. It has been suggested that excessive IL-1β promotes the aggregation of hIAPP during IDD progression and impairs the formation of nontoxic mature hIAPP monomers. Inflammation and ECM degradation play critical roles in the IDD process. Hyperactive ECM catabolism and inflammation have been characterized as the major phenotypes in degenerative IVD tissues^24^. Accumulating evidence has shown that mechanical compression affects IVD biology and that static compression promotes the progression of IDD^52^. Moreover, an overload of mechanical compression has been confirmed to induce the expression of proinflammatory cytokines and promote ECM catabolism in NP cells^52,53^. During the process of disc degeneration, IL-1β is upregulated, while IL-1Ra levels decrease, leading to hyperactive IL-1β signaling and accelerating the progression of IDD^27^. Park et al. reported that increased IL-1β levels impair the processing of pro-hIAPP and promote the deposition of immature hIAPP aggregates, which ultimately damages islet cell function^29^. In our ex vivo disc model, cotreatment with neutralizing IL-1β antibodies (nIL-1β) and hIAPP ameliorated IDD progression under static compression, while cotreatment with IL-1Ra (nIL-1Ra) antibodies and hIAPP accelerated the progression of IDD. Our results suggested that mechanical loading may promote the aggregation of hIAPP in NP tissues by regulating IL-1β/IL-1Ra signaling.

Interestingly, we observed that the overexpression of hIAPP decreased the level of IL-1β, reduced ECM degradation and attenuated apoptosis in NP cells. Consistent with our previous study, the overexpression of hIAPP exerted a mostly protective effect in NP cells in vitro^16^. This finding may be explained by the observation that hIAPP overexpression increases the formation of nontoxic hIAPP monomers and decreases the ratio of monomeric hIAPP aggregates. The number of hIAPP aggregates and the ratio of IL-1β/IL-1Ra both increased during compression-induced NP cell degeneration. However, the levels of IL-1β and IL-1Ra both decreased when NP cells directly overexpressed with hIAPP, indicating a balance in IL-1β signaling. Moreover, the blockade of IL-1β signaling with nIL-1β reduced the deposition and aggregation of hIAPP, which ameliorated the toxicity of hIAPP aggregates in the nIL-1β group compared to the control group. In contrast, the toxicity of hIAPP aggregates in NP cells was enhanced upon treatment with nIL-1Ra. These results suggested that hIAPP aggregates exacerbated the IDD process by accelerating the imbalance in IL-1β/IL-1Ra signaling.

Our results showed that hIAPP aggregates accelerated IDD progression and caused excessive cell apoptosis. During the IDD process, hIAPP aggregates may induce NP cell death by activating apoptotic signaling. The immature form of hIAPP, which presents as a toxic molecule that induces death signaling, including the FAS and caspase-3-dependent signaling pathways, is more likely to aggregate and be deposited^11^. A previous study showed that treatment with hIAPP induces FAS expression as well as the transcription of downstream genes^54^. The classical pathway is mediated by FAS receptor binding, resulting in the activation of the FAS-associated death receptor pathway. Caspase-8 is then activated and released into the cytoplasm, leading to the activation of caspase-9 and caspase-3 or the release of other pro-apoptotic factors, such as Bax, VDAC1, and cyto-C^54,55^. Moreover, the intrinsic apoptotic pathway can be elicited by various kinds of cellular stresses. The abnormal aggregation of hIAPP has been demonstrated to induce severe endoplasmic reticulum (ER) stress and oxidative stress as well as the unfolded protein response (UPR)^11,48^. Prolonged or severe UPR promotes the degradation of abnormal and normal cellular proteins and the expression of pro-apoptotic genes, ultimately causing cell apoptosis^56,57^. Inhibiting ER stress reduces the formation of hIAPP aggregates and ameliorates cellular dysfunction^9^. Therefore, severe cellular stresses, such as the mechanical loading-induced cellular stress used in our study, are potential mediators of hIAPP-induced NP cell apoptosis. In addition, several studies have reported that hIAPP aggregates enhance cell death by impairing autophagic flux; the activation of autophagy can attenuate the detrimental effects of hIAPP aggregates^13,58,59^. Autophagy is considered to be a cell self-protective mechanism, and impaired autophagic flux has been confirmed to promote the deposition of hIAPP aggregates^13^. Consequently, we hypothesize that hIAPP damages cells by impairing the balance of cellular protein processing, such as through autophagic degradation and ER stress, which accelerates the formation of hIAPP aggregates. In fact, the pathogenic mechanisms of hIAPP may be involved in the activation of the apoptotic signaling pathway, excessive cellular stresses, autophagic dysfunction, and more. A great deal of research is necessary to explain the underlying mechanisms.

The present study has certain limitations. First, the ex vivo model used in our experiments to mimic the IDD process has more advantages than monolayer cell culture, but it still does not fully represent in vivo conditions, especially during long-term IDD progression. Second, exogenous hIAPP treatment was used in our ex vivo experiments because mouse hIAPP cannot form aggregates spontaneously. However, the results would be more convincing if hIAPP transgenic mice has been used in this study. Third, the pathogenic mechanisms of hIAPP aggregates discussed in our study may represent only a small fraction of the mechanisms at play. More specific mechanisms of hIAPP aggregates in the progression of IDD still require further investigation.

In summary, we demonstrated that hIAPP aggregation in NP cells is associated with IDD progression. The deposition of the toxic hIAPP aggregates promoted NP cell apoptosis and ECM degradation by disturbing the balance of IL-1β/IL-1Ra signaling in ex vivo and in vitro systems. Therefore, methods aimed at inhibiting the formation of hIAPP aggregates in IVD tissues may prove beneficial for the treatment of IDD.

## Supplementary information

Supplementary Information
