# Author Correction: Targeting the IL-1β/IL-1Ra pathways for the aggregation of human islet amyloid polypeptide in an ex vivo organ culture system of the intervertebral disc

**DOI:** 10.1038/s12276-025-01468-3

**Published:** 2025-06-11

**Authors:** Xinghuo Wu, Zhiwei Liao, Kun Wang, Wenbin Hua, Xianzhe Liu, Yu Song, Yukun Zhang, Shuhua Yang, Cao Yang

**Affiliations:** Department of Orthopaedics, Union Hospital, Tongji Medical College, Huazhong University of Science and Technology, Wuhan, 430022 China

Correction to: *Experimental & Molecular Medicine* 10.1038/s12276-019-0310-7, published online 25 September 2019

After online publication of this article, the authors noticed an error in the Figure 3a and Figure 5 section.

During our check of the manuscript, we noticed an inadvertent duplication in Figure 3A (in the panel [FAS/Th-S in hIAPP group and Collagen II/Th-S in hIAPP+nIL-1β group]) and an inadvertent duplication in Figure 5I (in the panel [-nIL-1β in over-ctr and +nIL-1β in over-ctr group]). This occurred due to an oversight during figure assembly, where incorrect images were placed. This error does not affect the overall conclusions of the study but requires correction to ensure accuracy in data representation.
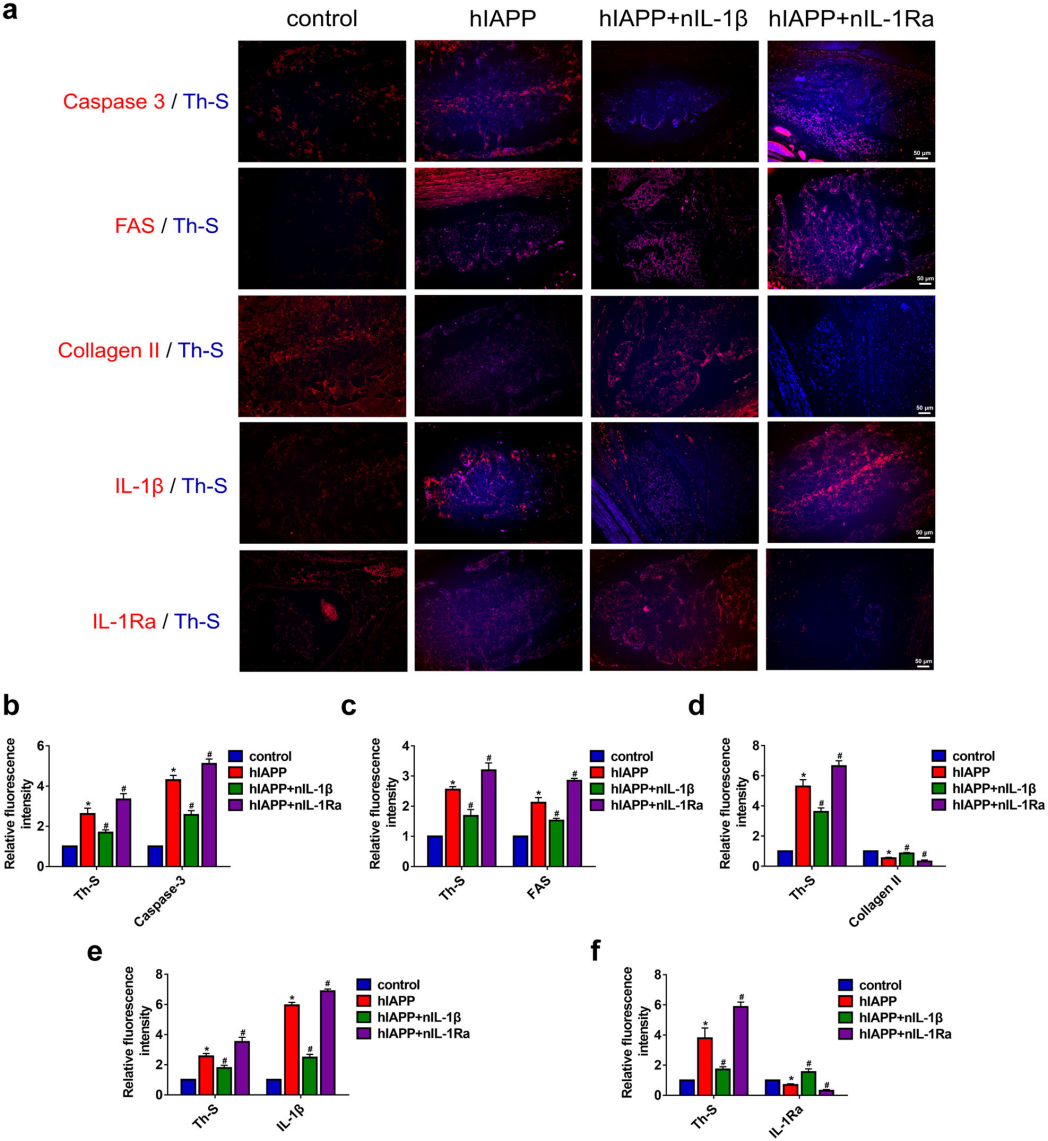

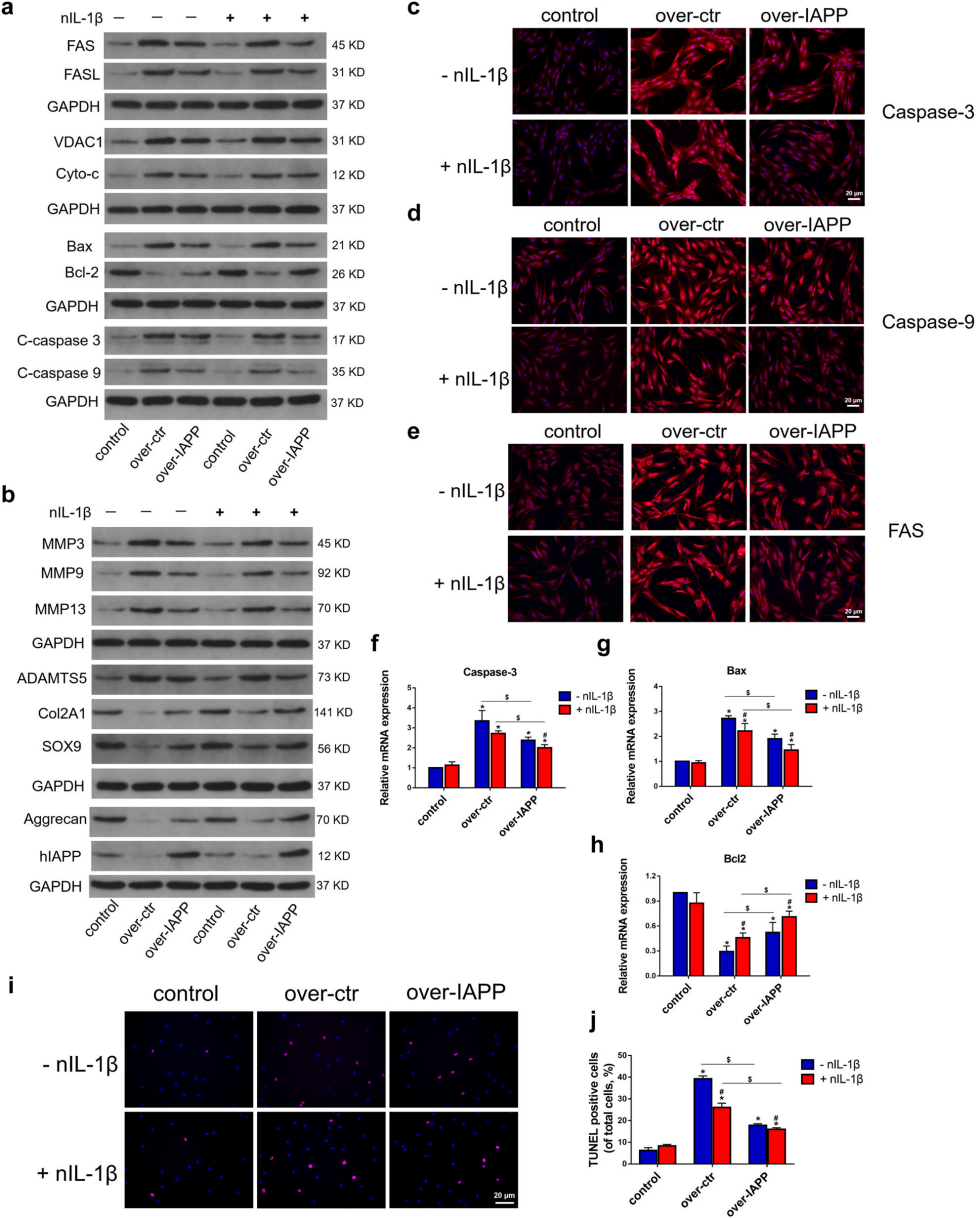


The authors apologize for any inconvenience caused.

The original article has been corrected.

