# Supplementary material for: Targeting the IL-1β/IL-1Ra pathways for the aggregation of human islet amyloid polypeptide in an ex vivo organ culture system of the intervertebral disc
Source: Exp Mol Med. 2019 Sep 25;51(9):1–16. doi: 10.1038/s12276-019-0310-7 (PMC6802624; doi:10.1038/s12276-019-0310-7)
Supplement: Supplementary file 1 — Supplementary Information [file 12276_2019_310_MOESM1_ESM.docx]

**Supplemental Information**

**Targeting IL-1β/IL-1Ra pathways for the Aggregation of Human Islet Amyloid Polypeptide in an ex vivo organ culture system of the intervertebral disc**

Xinghuo Wu^1^, Zhiwei Liao^1^, Kun Wang, Wenbin Hua, Xianzhe Liu, Yu Song, Yukun Zhang, Shuhua Yang, Cao Yang*


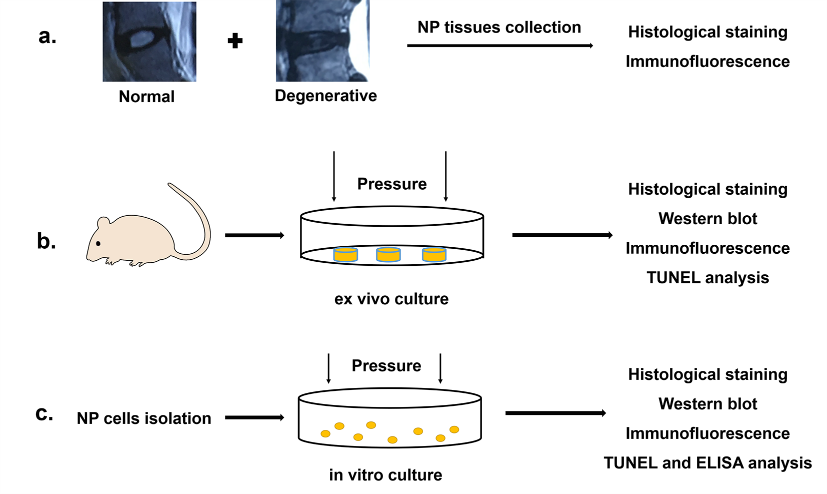


**Figure S1:** Schematic description of the experimental design. (a) NP tissues collected from scoliosis patients (normal) or patients with IDD disease (degenerative) were evaluated. (b) *Ex vivo* culture of whole IVD tissues were treated with static compression for 2 weeks. (c) NP cells were isolated and underwent a compression treatment for 7 days *in vitro*.


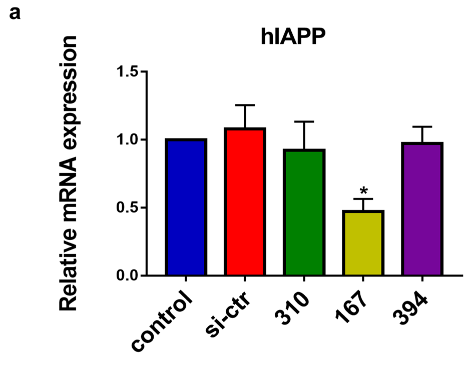


**Figure S2:** Three shRNA plasmids for hIAPP were designed and the efficiency of hIAPP knock-down was analyzed by RT-qPCR. To avoid off-target effects, there shRNA plasmids were all tested in NP cells experiments. Data were presented as the mean ± SD (n = 3). *P< 0.05 vs. control group.


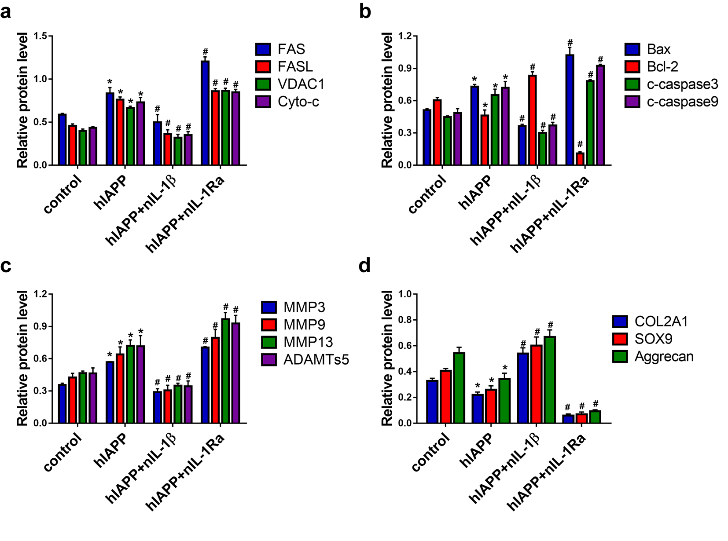


**Figure S3:** Quantification of protein levels. (a-b) The quantitative statistical analysis showed the protein expression levels of FAS, FASL, VDAC1, Cyto-c, Bax, Bcl-2, cleaved caspase-3, and caspase-9. GAPDH was used as an internal control. (c-d) The quantitative statistical analysis showed the protein expression levels of MMP3, MMP9, MMP13, ADAMTS5, COL2A1, SOX9, aggrecan, and hIAPP. GAPDH was used as an internal control. Data were presented as the mean ± SD (n = 3). *P< 0.05 vs. control group. ^#^P< 0.05 vs. nIL-1β non-treated group. ^$^P< 0.05 vs. corresponding group.


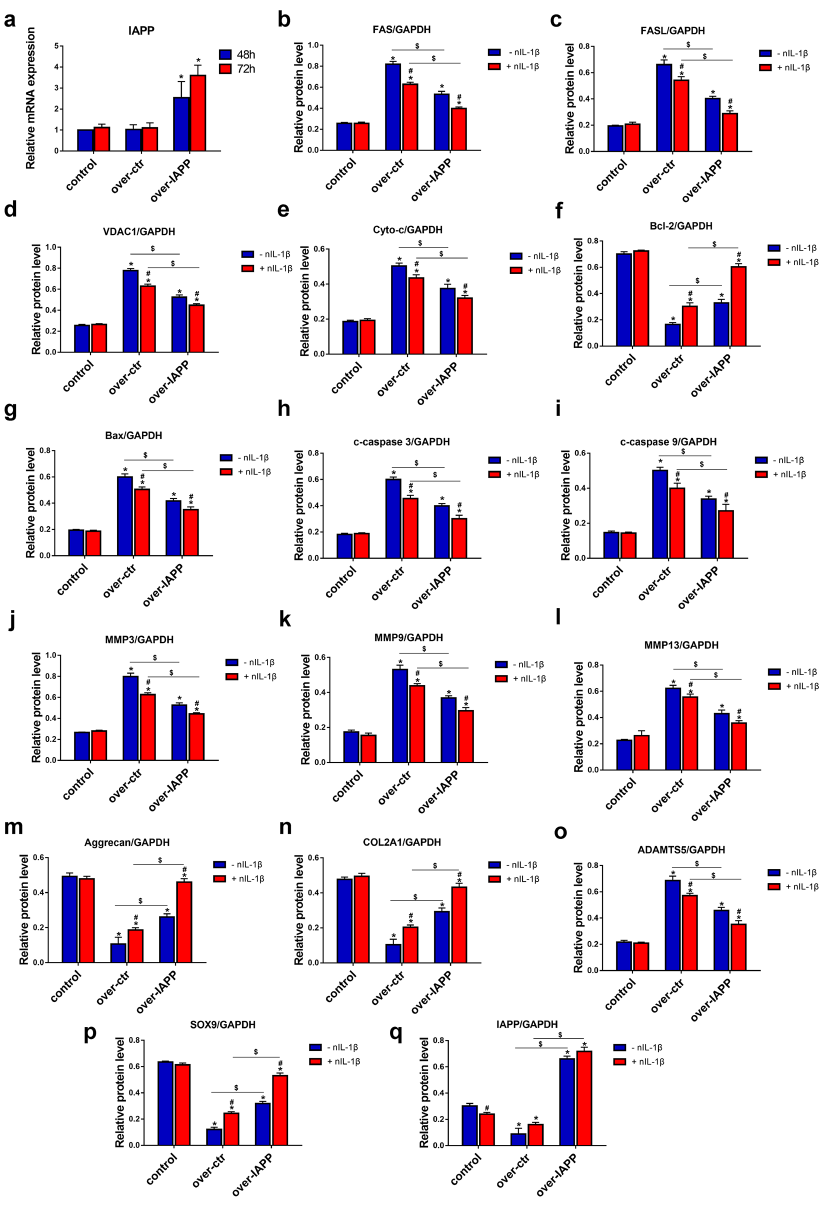


**Figure S4:** Effects of IL-1β neutralization on ECM remodeling and cell apoptosis in hIAPP-overexpressing NP cells. (a) The efficiency of hIAPP overexpression was analyzed by RT-qPCR at 48 h and 72 h after the transfection. (b-i) The quantitative statistical analysis showed the protein expression levels of FAS (b), FASL (c), VDAC1 (d), Cyto-c (e), Bcl-2 (f), Bax (g), cleaved caspase-3 (h), and caspase-9 (i). GAPDH was used as an internal control. (j-q) The quantitative statistical analysis showed the protein expression levels of MMP3 (j), MMP9 (k), MMP13 (l), aggrecan (m), COL2A1 (n), ADAMTS5 (o), SOX9 (p), and hIAPP (q). GAPDH was used as an internal control. Data were presented as the mean ± SD (n = 3). *P< 0.05 vs. control group. ^#^P< 0.05 vs. nIL-1β non-treated group. ^$^P< 0.05 vs. corresponding group.


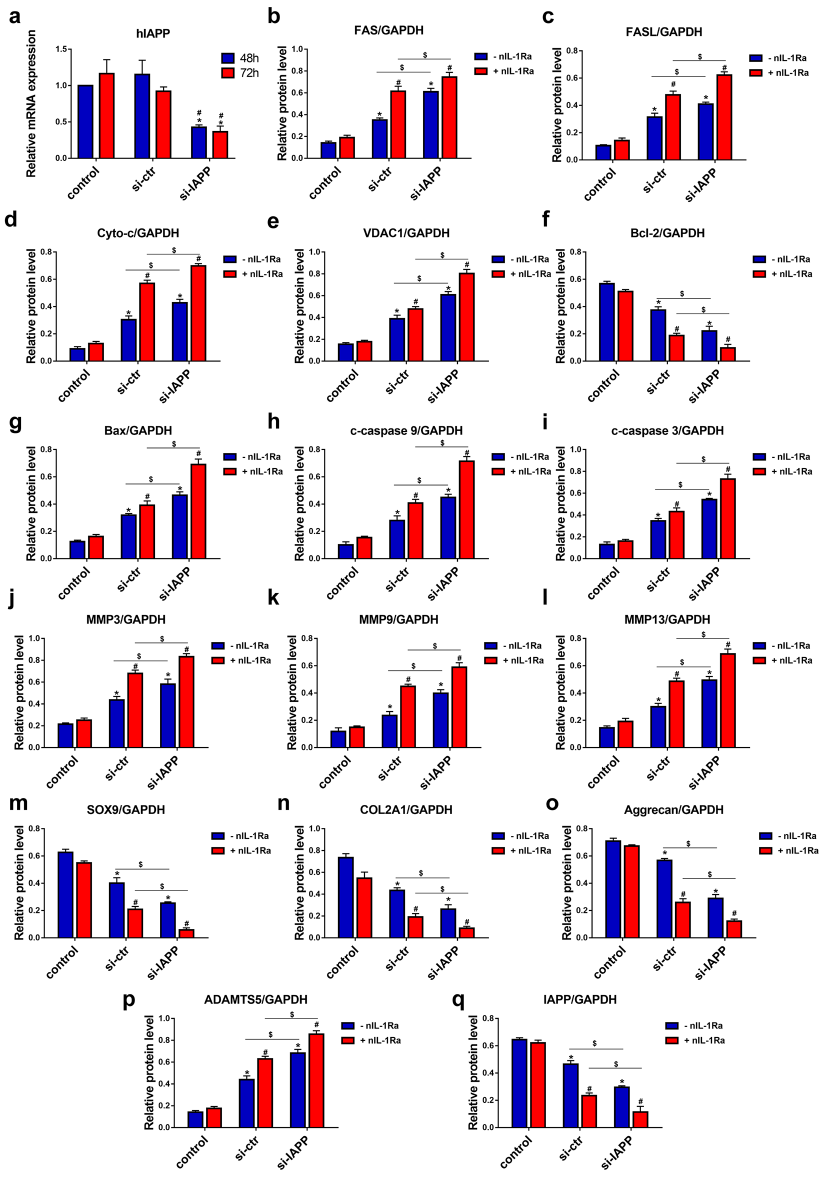


**Figure S5:** Effects of IL-1Ra neutralization on ECM remodeling and cell apoptosis in hIAPP-silencing NP cells. (a) The efficiency of hIAPP knock-down was analyzed by RT-qPCR at 48 h and 72 h after the transfection. (b-i) The quantitative statistical analysis showed the protein expression levels of FAS (b), FASL (c), Cyto-c (d), VDAC1 (e), Bcl-2 (f), Bax (g), cleaved caspase-9 (h), and caspase-3 (i). GAPDH was used as an internal control. (j-q) The quantitative statistical analysis showed the protein expression levels of MMP3 (j), MMP9 (k), MMP13 (l), SOX9 (m), COL2A1 (n), aggrecan (o), ADAMTS5 (p), and hIAPP (q). GAPDH was used as an internal control. Data were presented as the mean ± SD (n = 3). *P< 0.05 vs. control group. ^#^P< 0.05 vs. nIL-1β non-treated group. ^$^P< 0.05 vs. corresponding group.


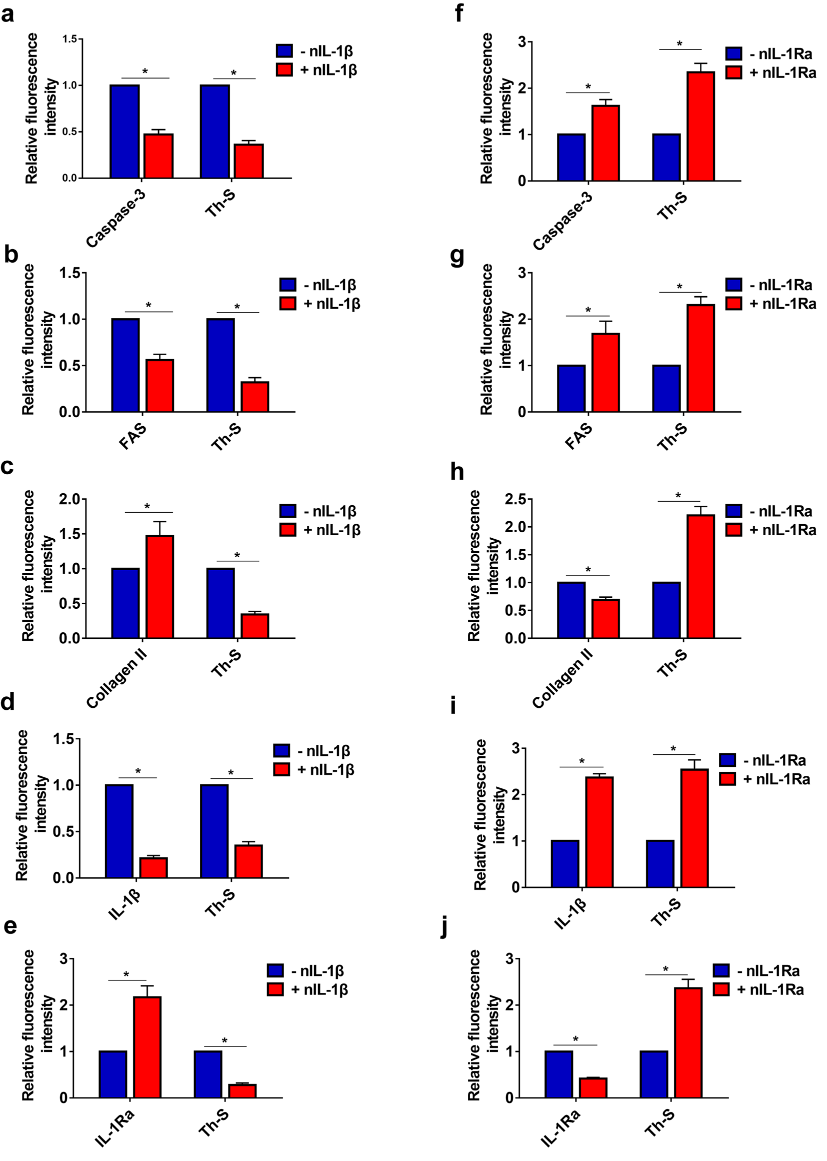


**Figure S6:** IL-1β/IL-1Ra signaling regulated ECM metabolism and cell apoptosis through the deposition of hIAPP aggregates in human NP cells. (a-e) Quantitative analysis of relative fluorescence intensity using Image-Pro Plus 6.0 for Th-S staining with immunofluorescence staining of caspase-3 (a), FAS (b), collagen II (c), IL-1β (d), and IL-1Ra (e). Data were presented as the mean ± SD (n = 3). *P< 0.05 vs. nIL-1β non-treated group. (f-j) Quantitative analysis of relative fluorescence intensity using Image-Pro Plus 6.0 for Th-S staining with immunofluorescence staining of caspase-3 (f), FAS (g), collagen II (h), IL-1β (i), and IL-1Ra (j). Data were presented as the mean ± SD (n = 3). *P< 0.05 vs. nIL-1Ra non-treated group.


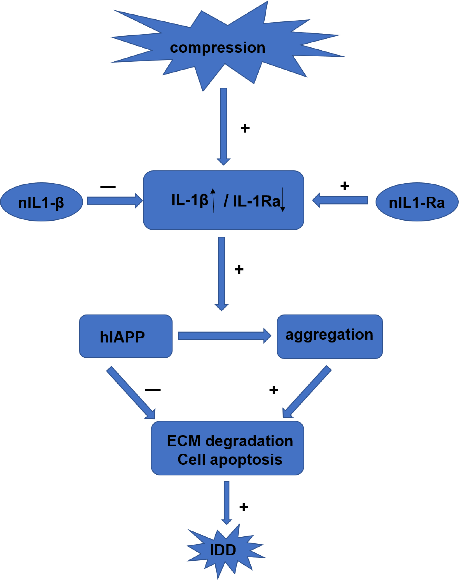


**Figure S7:** Schematic representation of the relationship between IL-1β/IL-1Ra signaling and hIAPP aggregation during compression-induced IDD. Compression-treatment resulted in an imbalance of IL-1β/IL-1Ra and increased level of IL-1β facilitated the formation of hIAPP. Deposition of hIAPP aggregates promoted the ECM degradation and excessive cell apoptosis. hIAPP, human islet amyloid polypeptide; IL-1β, interleukin-1β; nIL-1β, neutralizing antibody for IL-1β; IL-1Ra, the IL-1 receptor antagonist; nIL-1Ra, neutralizing antibody for IL-1Ra. ECM, extracellular matrix; IDD, intervertebral disc degeneration.

**Supplemental Table 1**

Samples information

| Number | Age (years) | Sex | Segment | Pfirrmann grade |
| --- | --- | --- | --- | --- |
| IS-1 | 17 | male | L2-3 | I |
| IS-2 | 22 | female | L2-3 | II |
| IS-3 | 15 | female | L1-2 | I |
| IS-4 | 20 | male | L3-4 | II |
| IS-5 | 18 | female | L2-3 | II |
| IDD-1 | 39 | female | L4-5 | IV |
| IDD-2 | 47 | female | L4-5 | V |
| IDD-3 | 54 | male | L5-S1 | V |
| IDD-4 | 32 | male | L4-5 | IV |
| IDD-5 | 37 | male | L3-4 | IV |

**Supplemental Table 2**

Primers of targeted genes

| Gene | Forward | Reverse | Size (bp) |
| --- | --- | --- | --- |
| IAPP | GCTACACCCATTGAAAGTC | GTTGTTGCTGGAATGAACT | 102 |
| Bax | AAGAAGCTGAGCGAGTGTCT | GTTCTGATCAGTTCCGGCAC | 236 |
| Caspase-3 | ACTGGACTGTGGCATTGAGA | GCACAAAGCGACTGGATGAA | 162 |
| Bcl-2 | GAAATCAAACAGAGGCCGCA | AGCGAGCATCCCCCAAAGTT | 192 |
| β-actin | AGCGAGCATCCCCCAAAGTT | GGGCACGAAGGCTCATCATT | 285 |

**Supplemental Table 3**

List of short hairpin RNA (shRNA) sequences

| shRNA | Sense | Anti-sense |
| --- | --- | --- |
| IAPP-homo-167 | GCUGCAAGUAUUUCUCAUUTT | AAUGAGAAAUACUUGCAGCTT |
| IAPP-homo-310 | GCAACAACUUUGGUGCCAUTT | AUGGCACCAAAGUUGUUGCTT |
| IAPP-homo-394 | GAGAGCCACUGAAUUACUUTT | AAGUAAUUCAGUGGCUCUCTT |
